# CD39 Expression in Peripheral CD4+ T Lymphocytes Is Associated With Disease Activity in Patients With Systemic Lupus Erythematosus

**DOI:** 10.1155/jimr/6676375

**Published:** 2026-03-02

**Authors:** Hao Jin, Lu Tang

**Affiliations:** ^1^ Technology Transfer Department, Tianjin Cancer Hospital Airport Hospital, Tianjin, China; ^2^ Division of Rheumatology, Tianjin First Central Hospital, Tianjin, China, tj-fch.com

**Keywords:** CD39, CD4-positive T lymphocytes, disease activity, systemic lupus erythematosus, Tregs

## Abstract

**Objective:**

Regulatory T cells (Tregs) and CD4‐positive T cells are crucial for the immunological control of systemic lupus erythematosus (SLE). As a crucial component of the adenosine metabolism pathway, CD39 influences the development and functionality of several immune system lymphocyte subsets, such as CD4‐positive T cells and Tregs. The purpose of this study was to examine the connection between the activity of SLE disease and the numbers of Tregs and CD4‐positive T lymphocytes, as well as the expression levels of the CD39 molecule on these cells.

**Methods:**

One hundred and eight SLE patients had peripheral blood drawn. The patients were split into two distinct categories: the SLE active group and the SLE low‐activity group, depending on the illness activity. Using flow cytometry, the proportions and absolute numbers of CD4‐positive T cells and Tregs, as well as the expression of CD39 on these cells, were measured. Their associations with SLE disease activity were examined. Last, CD39 was assessed as a possible biomarker for the activity of SLE illness.

**Results:**

Compared to the SLE low‐activity group, the SLE active group’s peripheral blood had a larger percentage and quantity of CD4‐positive T cells. In contrast, it was discovered that the SLE active group had fewer Tregs overall, both in terms of percentage and quantity, than the SLE low‐activity group. Compared to those in the SLE low‐activity group, patients in the SLE active group exhibited noticeably greater levels of CD39 expression in both Tregs and CD4‐positive T cells. Additionally, this study showed a favorable correlation between the percentage and absolute quantity of Tregs and the expression level of CD39 on Tregs. On the other hand, there is a negative correlation between the percentage and absolute quantity of CD4‐positive T cells and the expression level of CD39 on these cells. Furthermore, CD39 is a possible biomarker that could help in the identification of SLE disease activity, according to receiver operating characteristic (ROC) curve research.

**Conclusion:**

According to this study, CD4‐positive T cells’ surface CD39 molecule serves a similar purpose to Tregs’, namely immunological suppression. Therefore, a subpopulation of T cells with immunosuppressive properties is defined by CD4+CD39+ T lymphocytes, which may be a more accurate marker for differentiating disease activity in SLE.

## 1. Introduction

Systemic lupus erythematosus (SLE) exhibits diverse clinical manifestations, and most studies believe that abnormal immune regulation is the core factor in its pathogenesis. Defects in immune tolerance lead to exaggerated immune responses that damage organs, promoting the development of the disease [1]. Currently, the treatment of SLE has reached a bottleneck, and many researchers have begun to focus on immune regulatory therapy for SLE patients, aiming to address the immune imbalance and identify new therapeutic targets to overcome the current treatment limitations [2–4].

Some studies have indicated that systemic SLE is associated with an increase in CD4‐positive T cell activity and quantity [5–7]. By overproducing proinflammatory chemicals and cytokines, including interleukin‐6 (IL‐6) and tumor necrosis factor‐alpha (TNF‐α), these cells may trigger an inflammatory response [8–10]. Autoantibodies may be produced as a direct result of CD4‐positive T cells’ aberrant activation and proliferation, which can harm organs and self‐tissues. All things considered, CD4‐positive T cells are crucial to the development and course of SLE. Further investigation into the function and dysregulation of CD4‐positive T cells in SLE is critical for understanding the disease process and offering key hints for the development of successful treatment strategies [11].

As a subset of CD4‐positive T lymphocytes, regulatory T cells (Tregs) are essential for self‐immune tolerance. CD4+CD25+forkhead box P3 (FoxP3)+ is a typical phenotype of Tregs. Studies have shown that there are defects in the number and function of Tregs during the development of SLE, and the remission of SLE is positively correlated with the number and function of Tregs [1, 12].

Currently, research has shown that adenosine metabolism has a significant connection to the immune system, and CD39, as a key molecule in the adenosine metabolism pathway, plays a crucial role in the differentiation and function of various lymphocyte subsets within the immune system [13–15]. It has been reported that CD39 is one of the intrinsic immunosuppressive factors in the body, specifically expressed on the surface of FoxP3‐positive Tregs [16, 17]. It is a potential surface marker for Tregs and is involved in the immunosuppressive mechanisms of Tregs [18]. However, there have been relatively few studies on the CD39‐related pathway and its role in the diverse inhibitory functions of Tregs, both domestically and internationally [19, 20].

This study focused on examining the association between the proportions of CD4‐positive T lymphocytes and Tregs, as well as the expression levels of the CD39 molecule on these cells, and the SLE disease activity. The goal was to investigate the crucial function of the CD39 molecule on CD4‐positive T cells in immune regulation and provide new insights for effective clinical treatment strategies.

## 2. Materials and Methods

### 2.1. Materials

Following written informed permission, 108 SLE patients (ages 18–82) who were initially hospitalized at Tianjin First Central Hospital had peripheral blood samples taken. The Ethics Committee of Tianjin First Central Hospital gave its approval to the procedure. Prior to blood collection, none of the patients received any additional medical care. The inclusion and exclusion criteria were as follows. Inclusion criteria: (1) age between 18 and 65 years; (2) a confirmed diagnosis of SLE based on the 1997 revised classification criteria of the American College of Rheumatology (ACR);

(3) disease activity assessed using the SLE Disease Activity Index (SLEDAI). Patients were divided into two groups: the active group (SLEDAI > 9) and the low‐activity group (SLEDAI ≦ 9). Exclusion criteria: (1) history of malignancy; (2) prior treatment with rituximab or other biological agents; (3) use of high‐dose corticosteroids (>1.5 mg/kg/day) within the past month; (4) severe comorbidities including heart failure (New York Heart Association Class III or higher), renal insufficiency (creatinine clearance ≤30 mL/min), or hepatic dysfunction (ALT or AST ≥2 times the upper limit of normal); (5) active infections (e.g., hepatitis B, hepatitis C, HIV, and tuberculosis); (6) chronic infections; (7) pregnancy or lactation. All patients were fully informed about the objectives and procedures of the study and provided written informed consent before enrollment.

### 2.2. Methods

Peripheral blood samples were collected in EDTA tubes, and 200 µL of whole blood was used for flow cytometry staining and analysis. Following FcR blocking, cells were treated with 10 µL of antibodies that had been diluted properly before being rinsed with PBS. Anti‐human CD3‐PC7, anti‐human CD4‐PC5.5, anti‐human CD45‐Krome Orange (Beckman Coulter), anti‐human CD8‐APC‐Cy7, anti‐human CD39‐PE, and anti‐human CD25‐FITC (Biolegend) were among the antibodies utilized for cell surface staining in this investigation. For intracellular staining of FoxP3, cells were fixed and permeabilized using a fix/permeabilization kit (eBioscience) and stained with anti‐human FoxP3‐APC (Biolegend). Following surface and intranuclear staining procedures, the cells were washed and centrifuged. The resulting samples were then ready for loading onto the flow cytometer for analysis. The Navios flow cytometer (Beckman Coulter) was used for acquisition. To analyze the data, Beckman Coulter’s Kaluza software was used. Figure 1 illustrates the flow cytometry analysis procedure.

Figure 1Representative flow cytometry gating strategies for lymphocytes, CD3‐positive, CD4‐positive, CD8‐positive T lymphocytes, Tregs, and CD39 expression in the peripheral blood of SLE patients. (A) Representative flow cytometry plot from the SLE active group (*n* = 38) and (B) representative flow cytometry plot from the SLE low‐activity group (*n* = 70).

**Figure:**
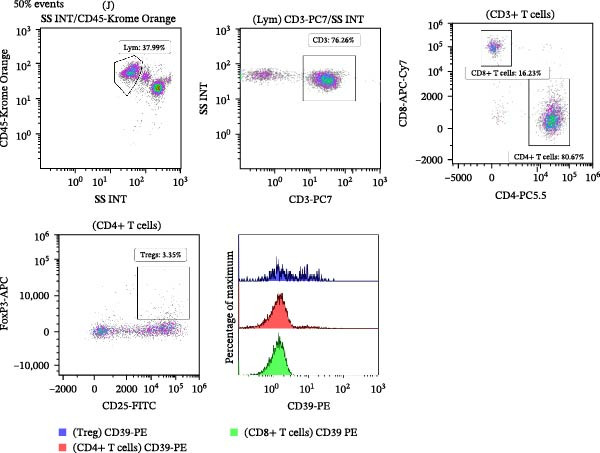
(A)

**Figure:**
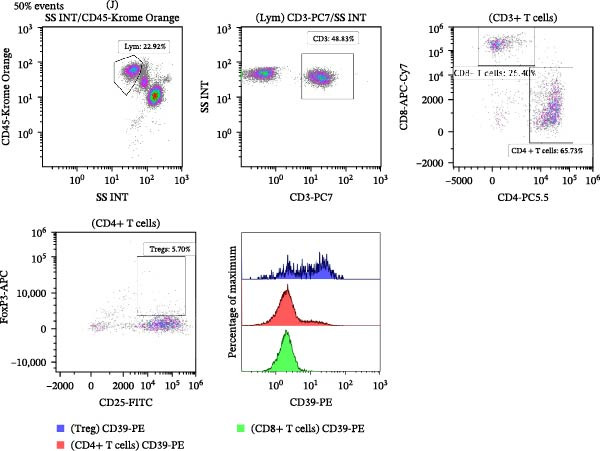
(B)

### 2.3. Statistical Analysis

All statistical analyses were conducted using SPSS Statistics 21 (IBM Corporation, NY, USA). The mean and standard deviation were employed to describe the numerical data since the Kolmogorov–Smirnov test indicated that the data had a normal distribution. To compare numerical data, the independent sample *t*‐test was employed. The nonparametric *t*‐test and Kruskal–Wallis test were used to determine statistical significance when the distribution matched the nonnormal distribution. Categorical variables were compared between groups using Fisher’s exact test, especially when the expected frequencies in any cell of the contingency table were below 5. The median and interquartile range were used to display the data. Graphpad Prism 9.5 was used to perform receiver operating characteristic (ROC) curve analysis, and the Youden index was used to identify the ideal cut‐off values in order to maximize both sensitivity and specificity. The threshold for statistical significance was set at *p*  < 0.05.

## 3. Results

### 3.1. The Association Between the SLE Disease Activity and Peripheral Blood Lymphocyte Subsets

This study enrolled 108 patients diagnosed with SLE. Patients were classified into two groups according to their disease activity scores, as determined by the SLEDAI: those with a score above 9 were classified as the SLE active group, and those with a score of 9 or below were classified as the SLE low‐activity group.

The clinical and laboratory characteristics of SLE patients in the low‐activity group and active group are summarized in Supporting Information 1: Table S1. There were no significant differences in age (55.31 ± 15.57 vs. 52.03 ± 17.54 years, *p* = 0.3188), gender distribution (*p* = 1.000, Fisher’s exact test), or serum immunoglobulin G (IgG) levels (1353 ± 474.7 vs. 1279 ± 479.7 mg/L,*p* = 0.4418) between the two groups. Although the percentage of anti‐dsDNA positivity was higher in the active group (18.4%) compared to the low‐activity group (10.0%), the difference did not reach statistical significance (*p* = 0.056, Fisher`s exact test). However, serum complement levels showed statistically significant differences. Patients in the low‐activity group had significantly higher levels of C3 (median: 89.60 [79.43–98.58] mg/L) and C4 (median: 19.95 [17.48–26.85] mg/L) compared to the active group (C3:70.24 28.57 mg/L, *p* < 0.0001; C4:16.13 ± 6.78 mg/L, *p* = 0.0003).

Four parameters were collected and analyzed, including the absolute counts and percentages of Tregs and CD4‐positive T lymphocytes in patients’ peripheral blood. The results of this analysis are presented in Table 1 and Figure 2. These findings provide valuable insights into the differences in T cell subsets between the active and remission phases of SLE. When comparing the percentage and numbers of these two T lymphocyte subsets, we observed a significant difference. The percentage and numbers of Tregs in the SLE active group were found to be decreased relative to the SLE low‐activity group (Figure 2A,C, *p* = 0.0311 ^∗^ and *p* = 0.0329 ^∗^, respectively), indicating a potential reduction in Tregs function during active SLE. Conversely, the percentage and absolute counts of CD4‐positive T lymphocytes in the peripheral blood of the SLE active group exhibited higher levels than the SLE low‐activity group (Figure 2B,D, *p* = 0.0099 ^∗∗^ and *p* = 0.0328 ^∗^, respectively). This suggests an elevated population of CD4‐positive T lymphocytes, which could result in elevated immune activation and disease activity in SLE. This finding suggests that in the SLE low‐activity group, there is an increased proportion and absolute count of Tregs, which corresponds to a decrease in CD4‐positive T lymphocytes.

Figure 2Analysis of the percentage and absolute number of CD4‐positive T lymphocyte subsets and Tregs in the peripheral blood of SLE patients. (A) Comparison of the percentage of Tregs in the peripheral blood of SLE active group and SLE low‐activity group; (B) comparison of the percentage of CD4‐positive T lymphocytes in the peripheral blood of SLE active group and SLE low‐activity group; (C) comparison of the number of Tregs in the peripheral blood of SLE active group and SLE low‐activity group; (D) comparison of the number of CD4‐positive T lymphocytes in the peripheral blood of SLE active group and SLE low‐activity group. The *p*‐value shown is obtained from the comparison between the indicated group by nonparametric *t* test.  ^∗^ *p* < 0.05 and  ^∗∗^ *p* < 0.01.

**Figure:**
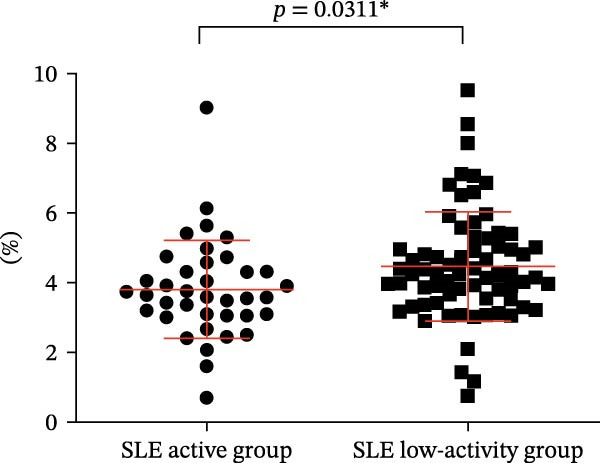
(A)

**Figure:**
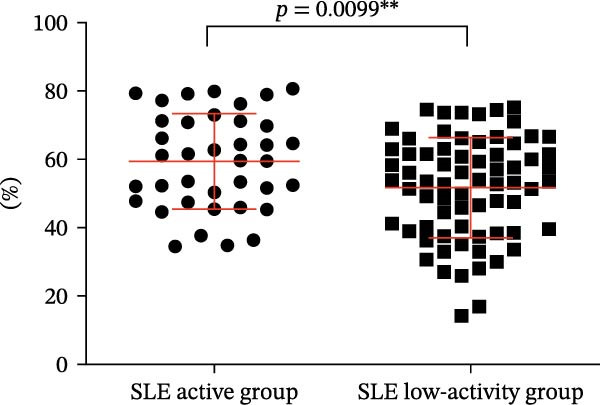
(B)

**Figure:**
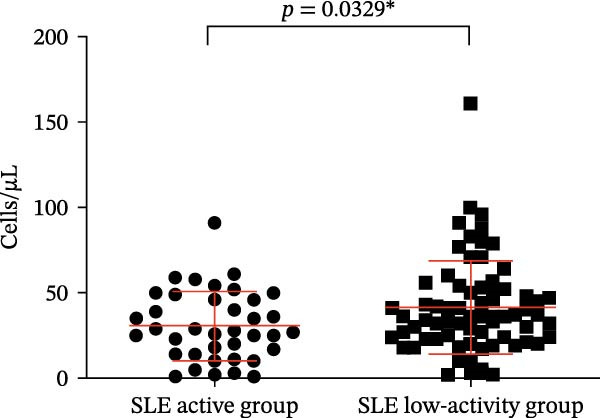
(C)

**Figure:**
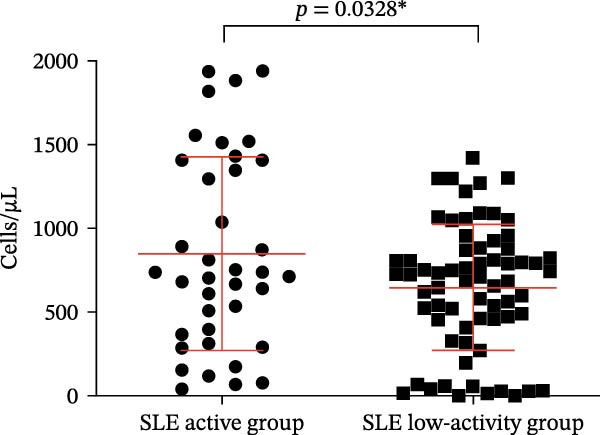
(D)

**Table 1:** Descriptive and analytical summary of Tregs, CD4‐positive T cells, and CD39 expression levels in the SLE active and low‐activity groups.

Variable	SLE active group	SLE remission group	*p*‐Value
Patients	38	70	—
The percentage of Tregs (%)	3.815 ± 1.411	4.260(3.505,5.103)	0.0311 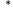
The percentage of CD4‐positive T cells (%)	59.46 ± 13.92	51.80 ± 14.75	0.0099 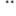
The number of Tregs (cells/µL)	30.63 ± 20.37	36.00 (23.50,52.50)	0.0329 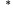
The number of CD4‐positive T cells (cells/µL)	848.5 ± 578.5	646.5 ± 377.2	0.0328 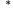
The percentage of CD39+ Tregs in Tregs (%)	14.20 (10.64,20.33)	35.64 ± 20.34	0.0016 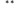
The percentage of CD39+ CD4+ T cells in CD4+ T cells (%)	3.040 (2.208,4.293)	10.820 (3.573,16.530)	0.0198 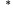

*Note:* Tregs, regulatory T cells.

Abbreviation: SLE, systemic lupus erythematosus.

^∗^ *p* < 0.05.

^∗∗^ *p* < 0.01.

The findings imply a potential imbalance and dysregulation within T lymphocyte subsets pertaining to SLE, which may contribute to the overall immune dysfunction observed in the disease. Further studies are essential to uncover the mechanisms and potential implications behind these observations.

### 3.2. The Association Between SLE Disease Activity and the CD39 Expression of T Subsets in Peripheral Blood

Adenosine is a crucial immunosuppressive regulator in the immune system, modulating immune responses through its influence on various immune cell types. CD39, an essential enzyme in the adenosine metabolic process, plays an essential role in this regulatory mechanism. Specifically, CD39 is known for its highest activity in Tregs, where its expression is regulated by FoxP3, a transcription factor specific to Tregs. This connection underscores the importance of CD39 in maintaining immune homeostasis and its potential impact on autoimmune diseases, notably SLE. This research investigates the levels of CD39 expression on both Tregs and CD4‐positive T lymphocytes and evaluates their correlation with SLE disease activity, aiming to determine whether the expression of CD39 on CD4‐positive T cells could confer similar immunosuppressive properties as seen in Tregs.

Our results revealed a significant relationship between CD39 expression in T cell subsets and SLE disease activity, as illustrated in Figure 3 and Table 1. Notably, patients in the active SLE group exhibited notably reduced percentages and numbers of Tregs compared to those in the low‐activity group, while the percentages and numbers of CD4‐positive T lymphocytes were higher in the active group. The low‐activity group exhibited a higher proportion of CD4+CD39+ T cells and CD39+ Tregs than the active group when analyzing CD39 expression (Figure 3A,B, *p* = 0.0016 and *p* = 0.0198, respectively). These observations imply that CD39 may help regulate immune responses and drive disease remission.

Figure 3(A) Comparison of the percentage of CD39+ Tregs in the peripheral blood of SLE active group and SLE low activity group; (B) comparison of the percentage of CD4+CD39+ T lymphocytes in the peripheral blood of SLE active group and SLE low activity group; (C) the correlation between the CD39 expression with the percentage of Tregs; (D) the correlation between the the CD39 expression with the absolute numbers of Tregs; (E) the correlation between the CD39 expression with the percentage of CD4‐positive T cells; (F) the correlation between the the CD39 expression with the absolute numbers of CD4‐positive T cells. The *p* value shown is obtained from the comparison between the indicated group by nonparametric *t* test.  ^∗^ *p* < 0.05 and  ^∗∗^ *p* < 0.01.

**Figure:**
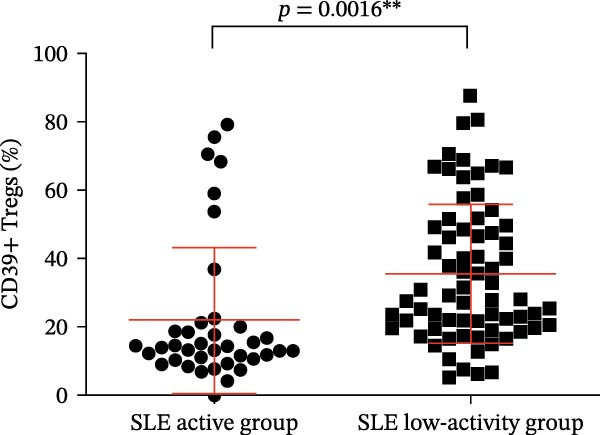
(A)

**Figure:**
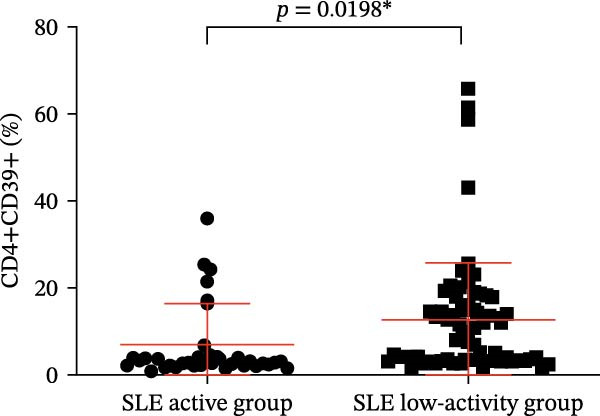
(B)

**Figure:**
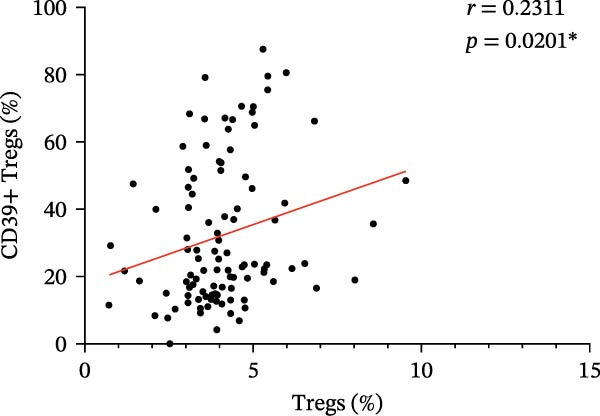
(C)

**Figure:**
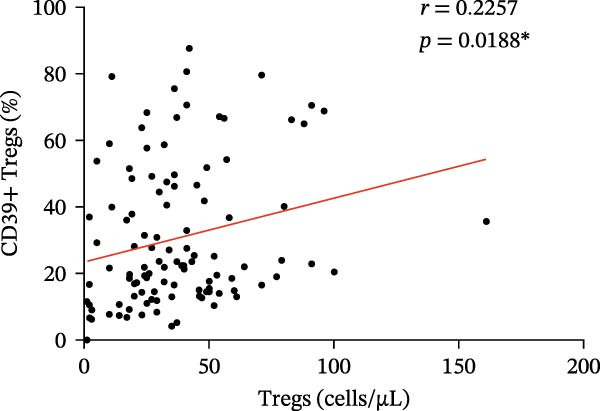
(D)

**Figure:**
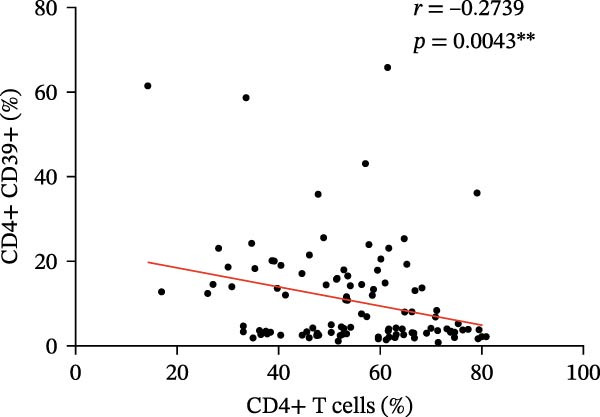
(E)

**Figure:**
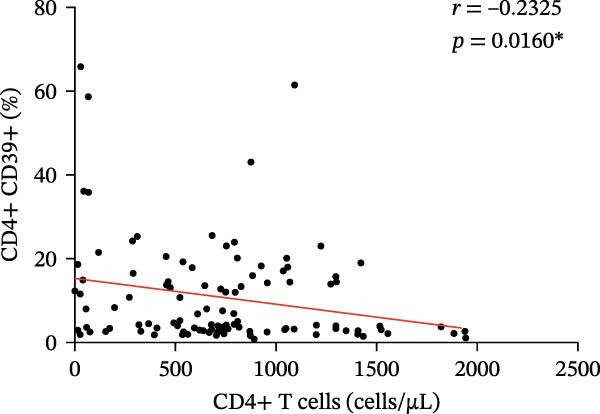
(F)

Further correlation analysis demonstrated that the proportion of CD39+ Tregs relative to total Tregs exhibited a positive relationship with both the percentage and absolute numbers of Tregs in the peripheral blood (Figure 3C,D, *r* = 0.2311, *p* = 0.0201 ^∗^ and *r* = 0.2257, *p* = 0.0188 ^∗^, respectively), suggesting a regulatory association between CD39 expression and Treg homeostasis. Additionally, we found that the proportion of CD4+CD39+ T cells demonstrated an inverse correlation with the proportion and absolute numbers of CD4+ T cells in SLE patients’ peripheral blood (Figure 3E,F, *r* = −0.2739, *p* = 0.0043 ^∗∗^ and *r* = −0.2325, *p* = 0.0160 ^∗^, respectively). The observed negative association indicates that as the level of CD39 expression on CD4‐positive T lymphocytes increases, the overall abundance of CD4‐positive T lymphocytes decreases.

To explore the potential of CD39+ T cell subsets as biomarkers of disease activity in SLE, we analyzed the correlation between the percentage of CD39+ Tregs and CD39+ CD4+ T cells and various clinical indicators, including anti‐dsDNA status, Ig levels (IgG), and complement levels (C3, C4). There was no statistically significant difference in the percentage of CD39+ Tregs or CD39+CD4+ T cells between anti‐dsDNA‐positive and ‐negative patients (*p*  > 0.05 for both as shown in Supporting Information 2: Figure S1A,B). Supporting Information 2: Figure S1C–H illustrate the correlations between CD39+ Tregs and CD39+CD4+ T cells and serum C3, C4, and IgG levels. Among these analyses, only CD39+ Tregs showed a significant positive correlation with serum C4 levels (Supporting Information 2: Figure S1E, *r* = 0.2408, *p* = 0.0125 ^∗^), indicating a potential link between CD39 expression in Tregs and complement activation. In contrast, no significant correlations were observed between CD39+ Tregs and C3 or IgG levels, nor between CD39+CD4+ T cells and C3, C4, or IgG levels (all *p*  > 0.05). These results suggest that while CD39+ Tregs may be partially associated with complement consumption, the overall correlation between CD39 expression and clinical parameters remains limited.

These results provide evidence supporting the hypothesis that CD39 expression on CD4‐positive T lymphocytes may induce immunosuppressive functions similar to those seen in Tregs, contributing to immune homeostasis and promoting disease remission in SLE patients. This suggests that the presence of CD4+CD39+ T cells may reflect a shift toward immune regulation, aiding in the suppression of excessive immune activity and facilitating the control of autoimmune responses.

### 3.3. Exploring CD39 as a Biomarker for Monitoring SLE Disease Activity

To evaluate the role of CD39 as a potential biomarker in the context of SLE disease activity, we conducted ROC curve analyses. The objective was to assess the diagnostic performance of various T cell subsets, including Tregs and CD4+ T cells, with and without CD39 expression, in predicting SLE disease activity.

Initially, we plotted ROC curves for the percentage of CD39+ Tregs, the percentage of Tregs, and the absolute number of Tregs, as shown in Figure 4A–D. These three parameters had corresponding area under the curve (AUC) values of 0.7564, 0.6468, and 0.5160. When compared to the percentage or absolute numbers of Tregs alone, the proportion of CD39+ Tregs showed the greatest AUC among these, indicating a higher discriminative ability to distinguish between active and remission states in SLE patients. The substantial improvement in AUC when including CD39 expression indicates that CD39 could enhance the diagnostic accuracy for assessing SLE disease activity.

Figure 4ROC curve analysis of Treg, CD39+ Treg, CD4+ T cells, and CD4+CD39+ T cell subsets in SLE diagnosis. (A) ROC curve showing the diagnostic accuracy of CD39+ Tregs (%), with an AUC of 0.7564; (B) ROC curve of Tregs (%), with an AUC of 0.6468; (C) ROC curve of Tregs (cells/µL), with an AUC of 0.5160; (D) combined ROC curve of CD39+ Tregs (%), Tregs (%), and Tregs (cells/µL), showing the merged diagnostic potential; (E) ROC curve of CD4+CD39+ T cells (%), with an AUC of 0.7078; (F) ROC curve of CD4+ T cells (%), with an AUC of 0.5628; (G) ROC curve of CD4+ T cells (cells/µL), with an AUC of 0.5301; (H) combined ROC curve of CD4+CD39+ T cells (%), CD4+ T cells (%), and CD4+ T cells (cells/µL), showing the merged diagnostic potential.

**Figure:**
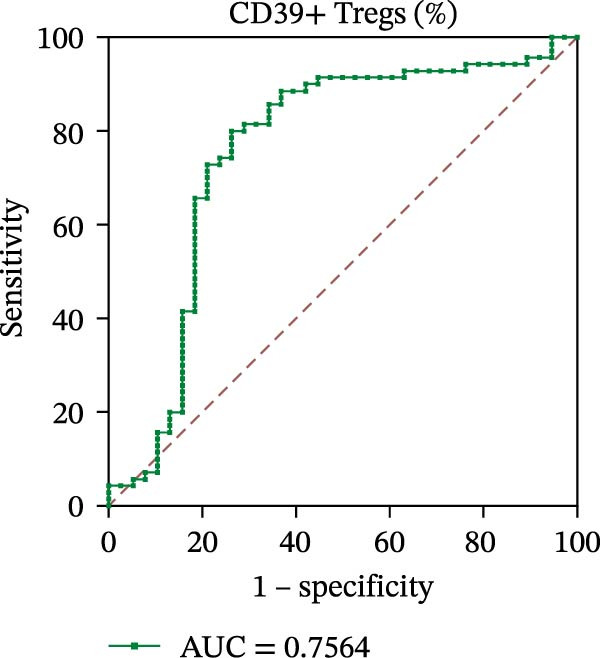
(A)

**Figure:**
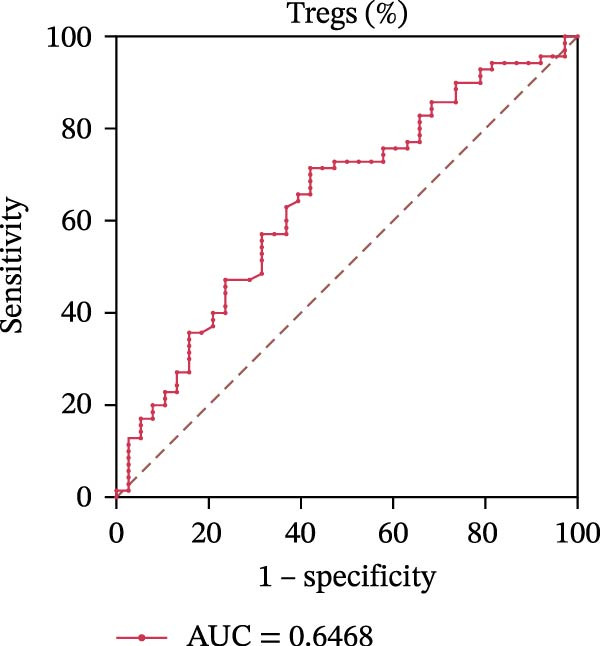
(B)

**Figure:**
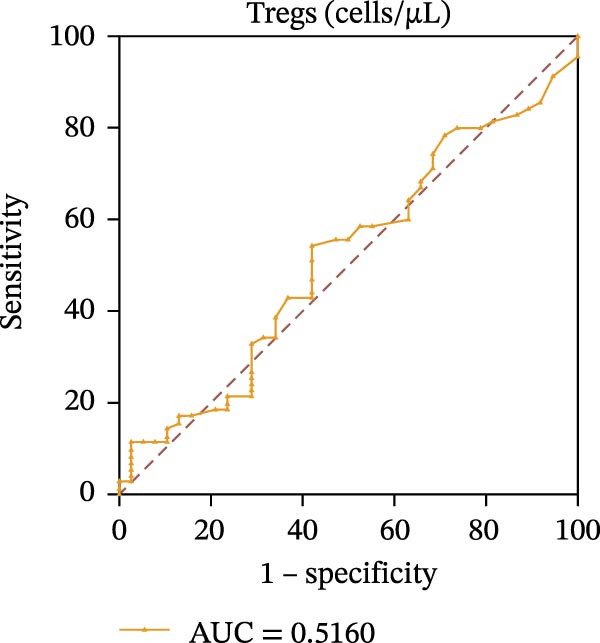
(C)

**Figure:**
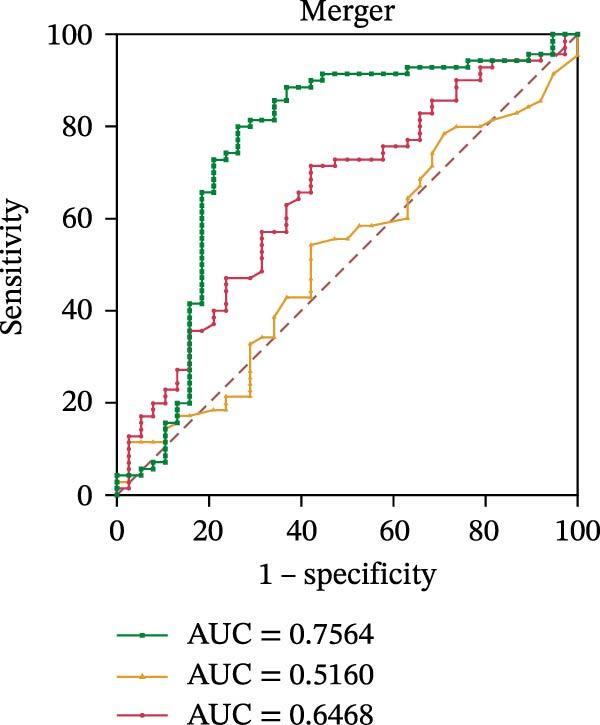
(D)

**Figure:**
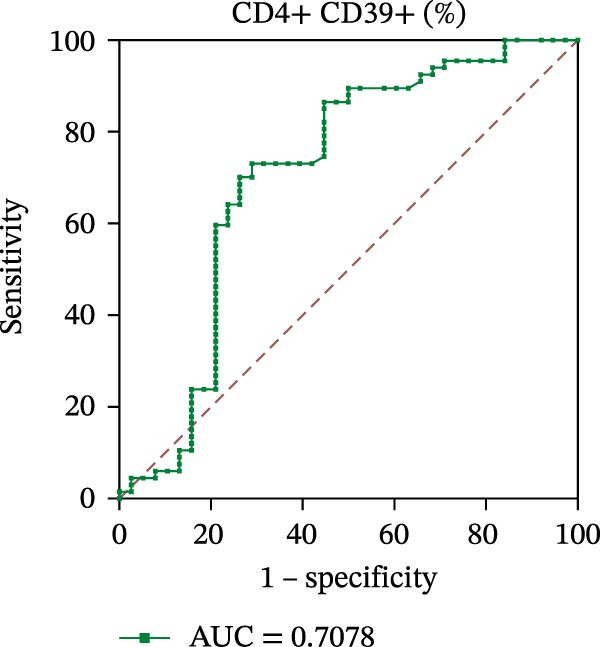
(E)

**Figure:**
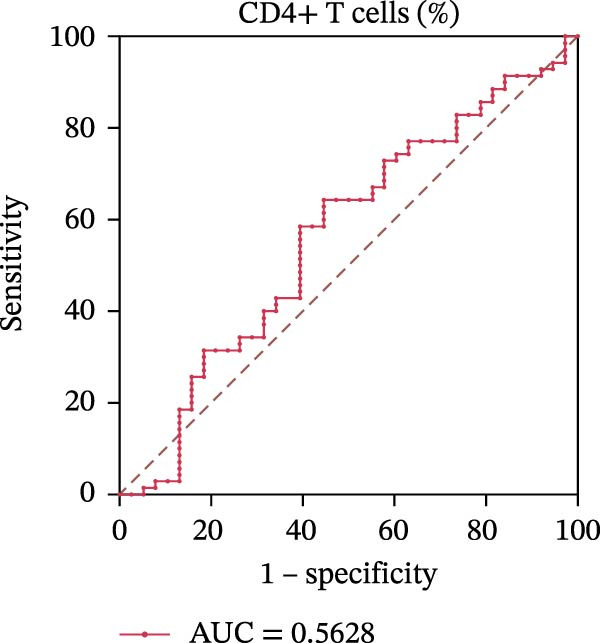
(F)

**Figure:**
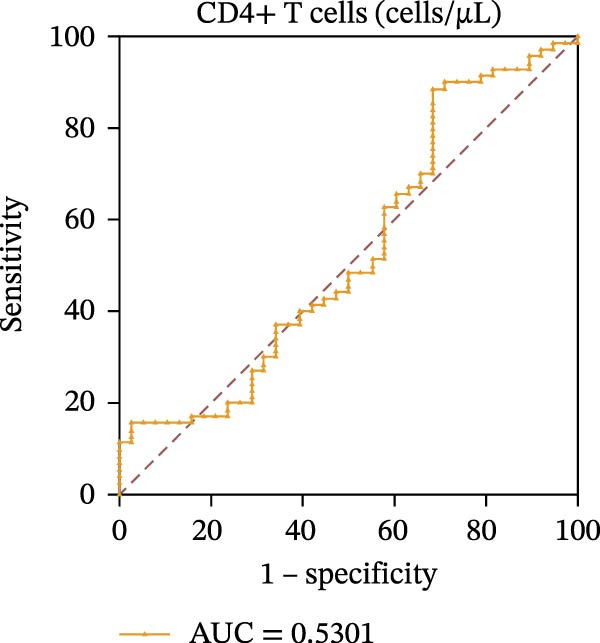
(G)

**Figure:**
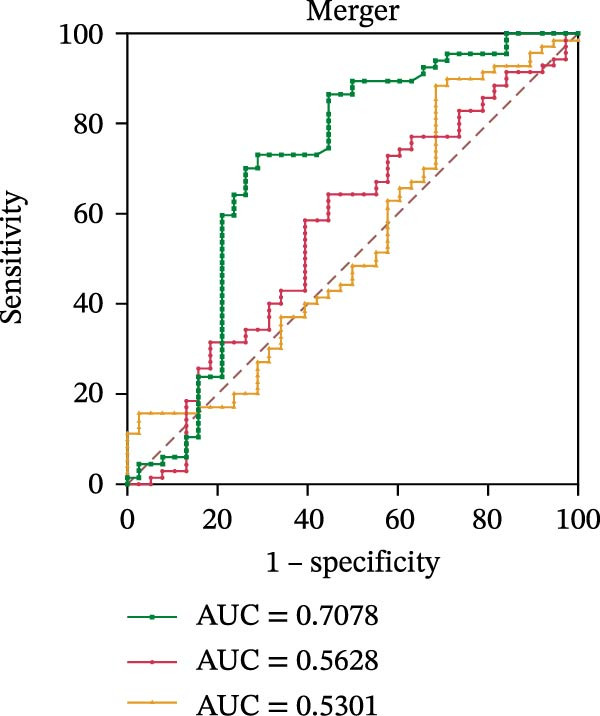
(H)

We then performed ROC curve analyses for CD4+ T cell subsets. The AUC values for the percentage of CD4+CD39+ T cells, the percentage of CD4‐positive T lymphocytes, and the absolute numbers of CD4‐positive T lymphocytes were 0.7078, 0.5628, and 0.5301, respectively (Figure 4E–H). Similar to the Tregs analysis, the percentage of CD4+CD39+ T cells showed a notable improvement in diagnostic performance, with a higher AUC compared to the percentage or absolute numbers of CD4+ T cells alone.

These findings suggest that CD39, as an immunosuppressive molecule, plays a significant role in modulating immune responses in SLE. Incorporating CD39 expression as part of the diagnostic assessment markedly enhances the accuracy in determining SLE disease activity. The inclusion of CD39+ Tregs and CD4+CD39+ T cells as biomarkers could provide a more precise tool for clinicians in diagnosing and tracking disease progression in SLE patients, potentially improving disease management and treatment outcomes.

## 4. Discussion

Currently, SLE disease activity is typically assessed using complex composite indices, such as the SLEDAI and the Systemic Lupus Activity Measure (SLAM) [21–24]. This increases the complexity of disease control and poses a significant challenge for clinicians in terms of rapid diagnosis and effective management. It is widely believed that Tregs contribute significantly to the onset and progression of SLE, and FoxP3 is the classical marker for Tregs. However, its nuclear localization limits its application in clinical research [2]. Currently, CD39 is also considered a surface marker for Tregs, indicating the extent of immune tolerance loss. The expression of CD39 on Tregs in SLE patients may potentially serve as a biomarker for lupus and an objective index for disease activity [8, 18, 25]. Studies by Loza et al. [26] have found defective expression of CD39 in SLE patients with low disease activity who were not treated with nonsteroidal immunosuppressants (NSISs), suggesting that the defect in CD39 expression could potentially serve as an indicator for identifying the disease in its early stages, prior to the onset of clinical symptoms. Early intervention measures that bypass this impairment may aid in reducing the progression of the disease. Furthermore, it was observed that some patients showed improved CD39 expression after treatment with steroids and immunosuppressants, indicating the special significance of CD39 expression in disease control [12, 27]. However, specific data on CD39 expression changes from inactive to active disease and after medication stabilization in the same subjects require further evaluation through longitudinal studies involving large sample sizes and high‐quality multicenter research [26, 28, 29].

Studies conducted abroad have found that adenosine receptor agonists can protect lupus mice from nephritis. In some lupus patients, adenosine deaminase activity increases in the blood, possibly related to reduced adenosine levels. In response to this change, the density of adenosine receptors on lymphocytes in lupus patients may increase [30–32]. These pathways can serve as novel pharmacological targets for lupus treatment by stimulating CD39 expression to enhance adenosine concentration and restore the immunosuppressive capacity of Tregs. It has been found that the number of CD39+ Tregs in patients significantly increases after treatment with glucocorticoids, suggesting that upregulation of CD39 expression may be one of the mechanisms underlying glucocorticoid treatment of SLE. Additionally, in SLE patients currently being treated with NSIS such as cyclophosphamide, azathioprine, mycophenolic acid, and methotrexate, the expression of CD39 does not significantly differ from that of healthy individuals [19, 33]. This indicates that NSIS may effectively treat SLE by inducing CD39 expression, amplifying the adenosine signaling pathway, increasing extracellular adenosine levels, and suppressing immune responses in target cells [26].

This study found a correlation between the progression of SLE disease activity and the quantities of CD4‐positive T cells and Treg cells. Subsequent research showed that the expression levels of the CD39 molecule on CD4‐positive T cells and Treg cells were also connected to SLE disease activity. Prior studies have shown that the CD39 molecule on the surface of Treg cells has a role in their ability to suppress the immune system. According to the findings of this study, the CD39 molecule on the surface of CD4‐positive T cells and Treg cells both contribute to immunological suppression. Therefore, a fraction of T cells with immunosuppressive properties is defined by CD4+CD39+ T cells.

This study depicts a novel hypothesis that the immunosuppressive pathway mediated by CD39, specifically its role in the adenosine metabolic pathway and its impact on both Tregs and CD4‐positive T lymphocytes, as shown in Figure 5. In Figure 5A, the role of CD39 on Tregs is highlighted. Treg surface‐expressed CD39 catalyzes the transformation of extracellular ATP (eATP) into AMP, which CD73 then transforms into adenosine. Adenosine, through its interaction with adenosine receptors (A1, A2A, A2B, and A3), mediates an immunosuppressive response by inhibiting the function of CD4‐positive T lymphocytes. Specifically, adenosine binding to A2A receptors increases intracellular cyclic AMP (cAMP) levels, leading to inhibition of effector T cell activity. This mechanism contributes to the expansion of Tregs and enhances their immunosuppressive activity, thereby maintaining immune homeostasis. Figure 5B illustrates the hypothesized transition pathway in which CD39 expressed on CD4‐positive T lymphocytes induces a similar adenosine‐mediated suppression pathway. As CD39 metabolizes eATP into AMP and then adenosine, it leads to increased local adenosine levels. This adenosine, by activating A2A receptors, inhibits CD4+ T lymphocyte function via cAMP signaling. Furthermore, this pathway may induce the transition of CD4+ T cells into Tregs, potentially through upregulation of FoxP3, a critical transcription factor for Treg differentiation. This transition promotes an increase in local immunosuppressive activity, contributing to immune regulation and suppression of overactive immune responses.

Figure 5A novel hypothesis that the immunosuppressive pathway mediated by CD39 depicted by this study. (A) CD39+ Tregs generate adenosine to suppress CD4+ T cells, promoting immune homeostasis and (B) CD39 on CD4+ T cells drives adenosine self‐suppression and potential Treg conversion.

**Figure:**
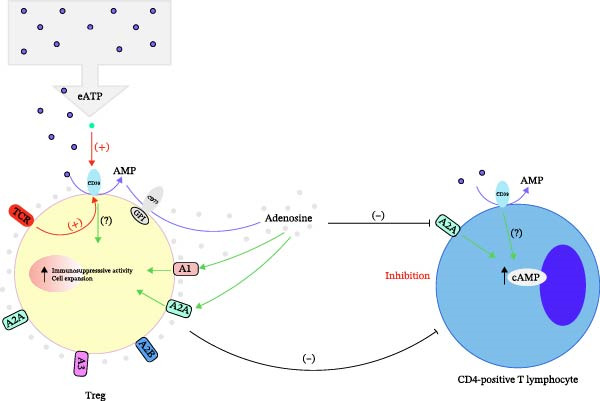
(A)

**Figure:**
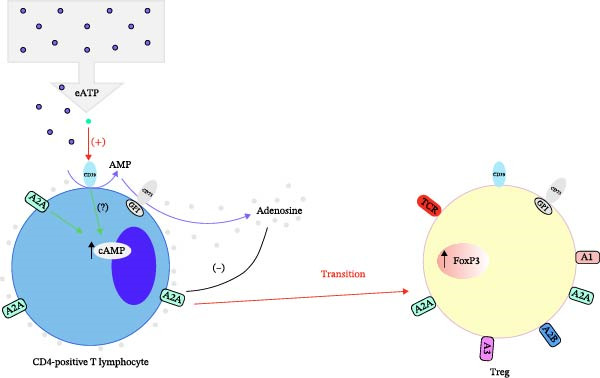
(B)

This research aims to explore the potential role of CD39 expression on CD4‐positive T cells in enhancing local adenosine metabolism, leading to their conversion into Tregs. The study’s findings suggest that CD4+CD39+ T cells possess similar immunosuppressive potential as Tregs, with CD39‐mediated adenosine metabolism playing a pivotal role in inducing this transition. This suggests that in autoimmune illnesses like SLE, where immunological suppression is essential for disease management, the CD4+CD39+ T cell fraction may play a major role in regulating immune responses.

This study also revealed that detecting CD39 expression can enhance the diagnostic accuracy of assessing SLE disease activity, offering new insights and potential biomarkers for future diagnostics. While Tregs have traditionally been effective in distinguishing between SLE active and low‐activity groups, CD39+ Tregs exhibit even greater diagnostic accuracy. Due to the inherent limitations of Tregs, such as their relatively low numbers in peripheral blood and the fact that their representative marker, FoxP3, is an intracellular nuclear marker, Treg detection can be challenging and prone to error. This study provides a new approach by highlighting the diagnostic potential of the CD4+CD39+ T cell subsets, which not only offer similar diagnostic value but are also easier to detect with greater accuracy.

In our study, both C3 and C4 levels were significantly decreased in the active group compared to the low‐activity group, which is consistent with the known pathophysiological mechanisms of active SLE and with clinical observations. To further explore the clinical relevance of CD39+ T cell subsets, we analyzed their correlation with key serological parameters. Interestingly, only CD39+ Tregs showed a significant positive correlation with serum C4 levels, while no significant associations were observed between CD39+ Tregs and C3, dsDNA, or Ig levels. Similarly, CD4+CD39+ T cells did not correlate significantly with any of the tested markers. This finding further supports the conclusion of our study: CD39 expression on Treg cells may serve as a more specific biomarker to distinguish disease activity status in SLE, especially in relation to complement activation. The unique association between CD39+Tregs and C4 highlights a potential immunoregulatory mechanism worth further investigation.

CD39 is a crucial component in the adenosine metabolism pathway, and its downregulation causes tissue damage and overactive immunological responses in SLE. The degree of immunological dysfunction linked to SLE may be correlated with the expression level of the CD39 molecule. Currently, the main treatment strategy for SLE is to suppress excessive immune responses in the body. CD39 can serve as a novel therapeutic target in clinical practice, acting as an endogenous anti‐inflammatory guardian against lupus and offering significant prospects for application. It provides new insights for disease treatment and disease monitoring.

## Author Contributions

Concept: Hao Jin and Lu Tang. Design: Hao Jin and Lu Tang. Supervision: Hao Jin and Lu Tang. Resources: Lu Tang. Materials: Lu Tang. Data collection and processing: Lu Tang and Hao Jin. Analysis and interpretation: Lu Tang and Hao Jin. Literature search: Lu Tang. Writing manuscript: Hao Jin. Critical review: Lu Tang and Hao Jin.

## Funding

This work was supported by grants from the Tianjin Binhai New Area Health Research Project (Grant 2024BWKZ09) and the National Natural Science Foundation of China (Grant 81602020).

## Disclosure

This manuscript has been previously posted as a preprint on Authorea [34].

## Ethics Statement

The authors are accountable for all aspects of the work in ensuring that questions related to the accuracy or integrity of any part of the work are appropriately investigated and resolved. The study was conducted in accordance with the Declaration of Helsinki (as revised in 2013). The study was approved by the Ethics Committee of Tianjin First Central Hospital. The approval number is the same as the National Natural Science Foundation of China (Number 81602020). The approval number is KY‐2024‐0059.

## Consent

The study was deemed exempt from institutional board approval, and patient informed consent was waived due to the retrospective nature and publicly available data source of the study.

## Conflicts of Interest

The authors declare no conflicts of interest.

## Supporting Information

Additional supporting information can be found online in the Supporting Information section.

## Supporting information


**Supporting Information 1** Table S1 provide detail clinical and laboratory characteristics of SLE patients in the low‐activity group and active group.

**Supporting Information 2** Figure S1 presents additional analyses exploring the association between CD39+ T cell subsets and clinical parameters in patients with SLE. This includes comparisons of CD39+ Tregs and CD4+CD39+ T lymphocytes between dsDNA(+) and dsDNA(‐) groups, as well as correlation analyses between CD39+ Tregs or CD4+CD39+ T cells and serum levels of C3, C4, and IgG. Among these analyses, a significant positive correlation was observed between CD39+ Tregs and C4 levels ( ^∗^ *p* < 0.05), while no other comparisons reached statistical significance.

## Data Availability

The data that support the findings of this study are available upon request from the corresponding author. The data are not publicly available due to privacy or ethical restrictions.
