# Supplementary material for: CD39 Expression in Peripheral CD4+ T Lymphocytes Is Associated With Disease Activity in Patients With Systemic Lupus Erythematosus
Source: J Immunol Res. 2026 Mar 2;2026:6676375. doi: 10.1155/jimr/6676375 (PMC13140799; doi:10.1155/jimr/6676375)
Supplement: Supplementary file 1 — Supporting Information 1 Table S1 provide detail clinical and laboratory characteristics of SLE patients in the low‐activity group and active group. [file JIMR-2026-6676375-s002.docx]

Supplement Table. Clinical and laboratory characteristics of SLE patients in the low activity group and active group.

|  | SLE active group | SLE low activity group | *P* value |
| --- | --- | --- | --- |
| Age | 52.03±17.54 | 55.31±15.57 | 0.3188 |
| Gender |  |  |  |
| Male | 3 | 5 | 1.000 |
| Female | 35 | 65 |  |
| dsDNA |  |  |  |
| Positive | 7 | 7 | 0.056 |
| Negative | 31 | 63 |  |
| IgG | 1279±479.7 | 1353±474.7 | 0.4418 |
| C3 | 70.24±28.57 | 89.60(79.43,98.58) | <0.0001**** |
| C4 | 16.13±6.780 | 19.95(17.48,26.85) | 0.0003*** |
|  |  |  |  |

SLE, Systemic Lupus Erythematosus; dsDNA, double-stranded DNA; C3, Complement Component 3; C4, Complement Component 4; **P*<0.05, ***P*<0.01, ****P*<0.001,*****P*<0.0001
