# Supplementary material for: CD39 Expression in Peripheral CD4+ T Lymphocytes Is Associated With Disease Activity in Patients With Systemic Lupus Erythematosus
Source: J Immunol Res. 2026 Mar 2;2026:6676375. doi: 10.1155/jimr/6676375 (PMC13140799; doi:10.1155/jimr/6676375)

Supplementary Figure

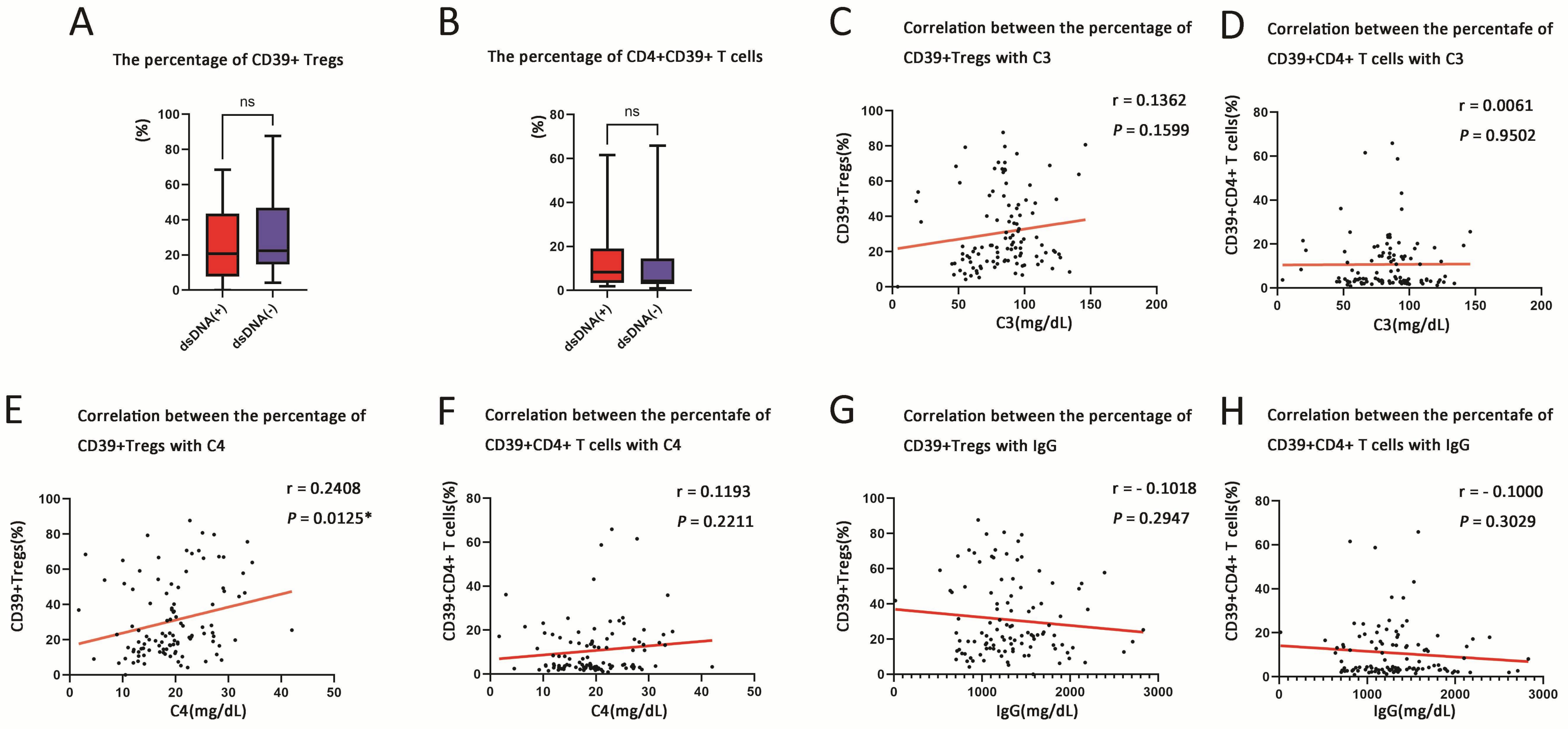

Supplement: Supplementary file 2 — Supporting Information 2 Figure S1 presents additional analyses exploring the association between CD39+ T cell subsets and clinical parameters in patients with SLE. This includes comparisons of CD39+ Tregs and CD4+CD39+ T lymphocytes between dsDNA(+) and dsDNA(‐) groups, as well as correlation analyses between CD39+ Tregs or CD4+CD39+ T cells and serum levels of C3, C4, and IgG. Among these analyses, a significant positive correlation was observed between CD39+ Tregs and C4 levels ( ∗ p < 0.05), while no other comparisons reached statistical significance. [file JIMR-2026-6676375-s001.pdf]
